# The relationship between early adulthood obesity and sarcopenic obesity among middle-aged and older adults in the United States

**DOI:** 10.3389/fpubh.2025.1609325

**Published:** 2025-09-01

**Authors:** Gang Cheng, Ying Zhou, Yan Wang, Chunguang Wang, Jianghong Xu

**Affiliations:** Department of Endocrinology, Dongfang Hospital, Beijing University of Chinese Medicine, Qinhuangdao Hospital (Qinhuangdao Hospital of Traditional Chinese Medicine), Qinhuangdao, Hebei, China

**Keywords:** sarcopenic obesity, early adulthood obesity, middle-aged and older adult, United States, NHANES

## Abstract

**Objective:**

This study aimed to explore the relationship between early adulthood obesity and sarcopenic obesity (SO) among middle-aged and older adults in the United States.

**Methods:**

This retrospective study was conducted involving adults aged 50–69 years in the United States. Data were extracted from the National Health and Nutrition Examination Survey (NHANES) during the periods 1999–2006 and 2011–2018. Height and weight at the age of 25 years were measured. Body mass index (BMI) at the age of 25 years (BMI_25_) was calculated. Healthy weight, overweight, and obesity at the age of 25 years (healthy weight_25_, overweight_25_, and obesity_25_) were defined as BMI_25_ 18.5 to less than 25 kg/m^2^, 25 to less than 30 kg/m^2^, and 30 kg/m^2^ or greater, respectively. SO was determined by dividing appendicular skeletal muscle mass by weight (ASM/Wt) and percentage of fat mass [FM (%)].

**Results:**

The prevalence of SO was 5.4, 8.5, and 16.5% in healthy weight_25_, overweight_25_, and obesity_25_ groups, respectively. After adjusting for confounding factors, the prevalence of SO in the overweight_25_ group and the obesity_25_ group was 1.161 times (95%CI: 0.898–1.500, *p* = 0.254) and 2.286 times (95%CI: 1.651–3.165, *p* < 0.001) higher, respectively, compared to the healthy weight_25_ group.

**Conclusion:**

This study demonstrates that obesity in early adulthood is closely associated with an increased risk of sarcopenic obesity among middle-aged and older adults.

## Introduction

Sarcopenic obesity (SO) is defined as the simultaneous presence of excessive fat mass and reduced muscle mass in an individual. While sarcopenia and obesity are independently associated with an increased risk of multimorbidity, their coexistence in the form of SO results in an even higher risk (1). Individuals with SO are more susceptible to frailty and exhibit a greater likelihood of falls and elevated rates of non-vertebral fractures in older adults (2, 3). Compared to non-SO individuals, those with SO also show a higher prevalence of cognitive impairment, coronary artery disease, and dyslipidemia (4). A meta-analysis of prospective cohort studies reported that SO was associated with a 24% increased risk of all-cause mortality (5). An updated meta-analysis further confirmed that SO is a significant predictor of all-cause mortality in middle-aged and older adults, regardless of whether they are community-dwelling or hospitalized (6). SO presents a substantially higher risk of multimorbidity than either sarcopenia or obesity alone. This increased risk is due to synergistic biological mechanisms that intensify inflammation, insulin resistance, and physical dysfunction (1, 7, 8).

Body mass index (BMI) is a widely used clinical indicator for assessing obesity. Multi-cohort studies have demonstrated that BMI-defined obesity is closely associated with 21 non-overlapping cardiometabolic, digestive, respiratory, neurological, musculoskeletal, and infectious diseases in adults (9). Early-life exposure to obesity also influences health outcomes in later life. Previous studies consistently indicate that individuals who are overweight or obese in early adulthood are at an increased risk of remaining overweight or obese in later life (10). Obesity, in conjunction with aging, may exacerbate the decline in muscle mass and function (11). Early-onset obesity has been linked to an increased risk of mobility limitations and cognitive decline in old age (12–15). Moreover, obesity during early adulthood is associated with a higher risk of mortality in later life (16).

However, the relationship between early adulthood obesity and the subsequent development of SO remains underexplored. Non-pharmacological interventions have been shown to effectively alleviate the clinical symptoms and signs associated with SO (17). Thus, early identification of individuals at risk for SO is essential to prevent disease progression and reduce the burden of comorbidities. Investigating the association between early adulthood obesity and SO in middle-aged and older populations may facilitate the early identification of high-risk individuals and provide a critical window for timely intervention.

The National Health and Nutrition Examination Survey (NHANES), conducted by the National Center for Health Statistics (NCHS), is a comprehensive program designed to assess the health and nutritional status of adults and children in the United States. The NHANES dataset includes information on body weight and height at age 25, along with body composition measurements in middle-aged and older adults. In the present study, we utilized the NHANES database to analyze the relationship between early adulthood obesity and SO in middle-aged and older adults in the United States, with the aim of identifying high-risk populations at an earlier stage.

## Methods

### Study design

This is a retrospective cohort study that analyzed data obtained from the NHANES. All NHANES data collection protocols were approved by the National Center for Health Statistics Research Ethics Review Board. Eight cycles (1999–2006 and 2011–2018) of data were included in the analysis for this study. Body composition was measured among subjects aged 8–69 years. Information about height and weight at age 25 years was collected among subjects aged over 50 years. Therefore, this study included middle-aged adults aged 50–69 years. The exclusion criteria were as follows: (1) missing data on body composition, such as appendicular skeletal muscle mass and percentage of fat mass; (2) missing data on height and weight at age 25 years; (3) body mass index (BMI) at age 25 years < 18.5 kg/m^2^; and (4) missing covariate data. The NHANES protocol received approval from the NCHS Research Ethics Review Board (Protocol #98-12, Protocol #2005-06, Protocol #2011-17, and Protocol #2018-01)^1^ and was performed in accordance with the Declaration of Helsinki. To ensure the protection of the participants’ rights, NHANES has obtained informed written consent from all the individuals involved in the study.

### Definition of overweight and obesity at age 25 years

The levels of height (inches) and weight (pounds) at age 25 years were obtained. They were then converted to height (meters = inches * 0.0254) and weight (kilograms = pounds * 0.4536). BMI at age 25 years (BMI_25_) is calculated as weight at age 25 years (weight_25_ in kilograms) divided by the square of height at age 25 years (height_25_ in meters). Healthy weight, overweight, and obesity at age 25 years (healthy weight_25_, overweight_25_, and obesity_25_) were defined as BMI_25_ of 18.5 to less than 25 kg/m^2^, 25 to less than 30 kg/m^2^, and 30 kg/m^2^ or greater, respectively.^2^

### Definition of sarcopenic obesity

Skeletal muscle mass was measured by dual-energy x-ray absorptiometry (DXA). Whole-body scans were acquired using Hologic QDR-4500A fan-beam and Hologic Discovery model A densitometers (Hologic, Inc., Bedford, MA, USA), using software versions 8.26:a3*, v12.4, and Apex 3.2, respectively. Appendicular skeletal muscle mass (ASM) was assessed as the sum of the muscle mass of the upper and lower limbs. ASM divided by weight (ASM/Wt) was used to calculate: ASM/Wt (%) = ASM (kg)/weight (kg)*100. Percentage of fat mass (FM (%)) was calculated as the ratio of DXA whole-body fat mass (g) to DXA whole-body total mass (g) *100.

SO was defined using the European Society for Clinical Nutrition and Metabolism (ESPEN) and the European Association for the Study of Obesity (EASO) criteria (18). Sarcopenia was defined as ASM/Wt < 25.7% for men and < 19.4% for women (19). The relationship between percent body fat and BMI differs among ethnic groups (20); therefore, ethnicity-specific cutoffs are applied for fat mass in this study. Obesity was defined as FM% > 27% (Non-Hispanic Black) and > 29% (Other Race) for men and > 39% (Non-Hispanic Black) and > 41% (Other Race) for women in adults aged < 60 years and FM% > 29% (Non-Hispanic Black) and > 31% (Other Race) for men and > 41% (Non-Hispanic Black) and > 43% (Other Race) for women in adults aged ≥ 60 years (21). SO was defined as having sarcopenia and obesity simultaneously.

### Muscle function

An observed timed 20-foot walk was used to assess functional limitations in the 1999–2000 and 2001–2002 cycles. The 20-foot walk was timed using a handheld stopwatch. The examinee was asked to walk at their usual pace. The muscle strength test component measured grip strength using a handgrip dynamometer in the 2011–2012 and 2013–2014 cycles. The combined grip strength was calculated as the sum of the largest reading from each hand and expressed in kilograms. Muscle quality index (MQI, kg/kg) was defined as the ratio of combined grip strength (kg) to ASM (kg) (22).

### Covariates

Sociodemographic covariates included age, gender, race, and family poverty income ratio (PIR). Smoking history was defined as follows: never (smoked fewer than 100 cigarettes in a lifetime), former smoker (smoked ≥ 100 cigarettes in a lifetime but does not currently smoke), or current smoker (smoked ≥ 100 cigarettes in a lifetime and currently smokes). Dietary energy and protein intake were assessed using the 24-h dietary recall method. Physical activity was defined as participating in moderate or vigorous activities. Height (meters) and weight (kilograms) measurements were collected, and BMI (kg/m^2^) was calculated at the time of the survey. Chronic disease status and medication use were also evaluated. Diabetes was defined based on the following criteria: fasting glucose levels of ≥126 mg/dL, a hemoglobin A1c level of ≥6.5%, a history of diabetes, or the current use of diabetes medications. Hypertension was defined as having systolic blood pressure (SBP) of ≥140 mmHg, diastolic blood pressure (DBP) of ≥90 mmHg, or currently taking medications for hypertension. Cardiovascular disease was determined based on the affirmative responses to the following question: “Has a doctor ever told you that you had congestive heart failure, coronary heart disease, angina/angina pectoris, a heart attack, or a stroke?” Cancer was determined based on an affirmative response to the question: “Has a doctor ever told you that you had cancer or malignancy?” The total number of prescription medications was also recorded.

### Statistical analyses

Analyses were performed using Stata software (version 16.0) (STATA Corporation, TX, USA). Quantitative data were expressed as means with standard deviations. The three groups were compared using analysis of variance (ANOVA). Categorical data were compared using the chi-squared test. Logistic regression models were used for modeling relationships between obesity status at age 25 and SO among middle-aged and older adults. Model 1: This was a univariate logistic regression analysis. Model 2: This model was further adjusted for age, gender (Men = 1, Women = 2), ethnicity (Non-Hispanic White = 0, Non-Hispanic Black = 1, and Other Race = 2), Family poverty income ratio (PIR), smoking history (Never = 0, Former smoker = 1, and Current smoker = 2), energy intake, protein intake, physical activity (No = 0 and Yes = 1), diabetes (No = 0 and Yes = 1), hypertension (No = 0 and Yes = 1), cardiovascular disease (No = 0 and Yes = 1), cancer (No = 0 and Yes = 1), and number of prescription medicines as covariates. Model 3: This model is further adjusted for BMI at the time of survey (BMI < 25 kg/m^2^ = 0 and BMI ≥ 25 kg/m^2^ = 1) as covariates. Variance inflation factors (VIFs) were calculated, and VIFs above 10 indicated multicollinearity. Survey weights, strata, and primary sampling units were also applied to the analyses to account for the NHANES complex and multi-stage survey design. The analysis was also stratified by gender, age, race, and BMI at the time of the survey. *p* < 0.05 was considered statistically significant.

## Results

**Figure 1:**
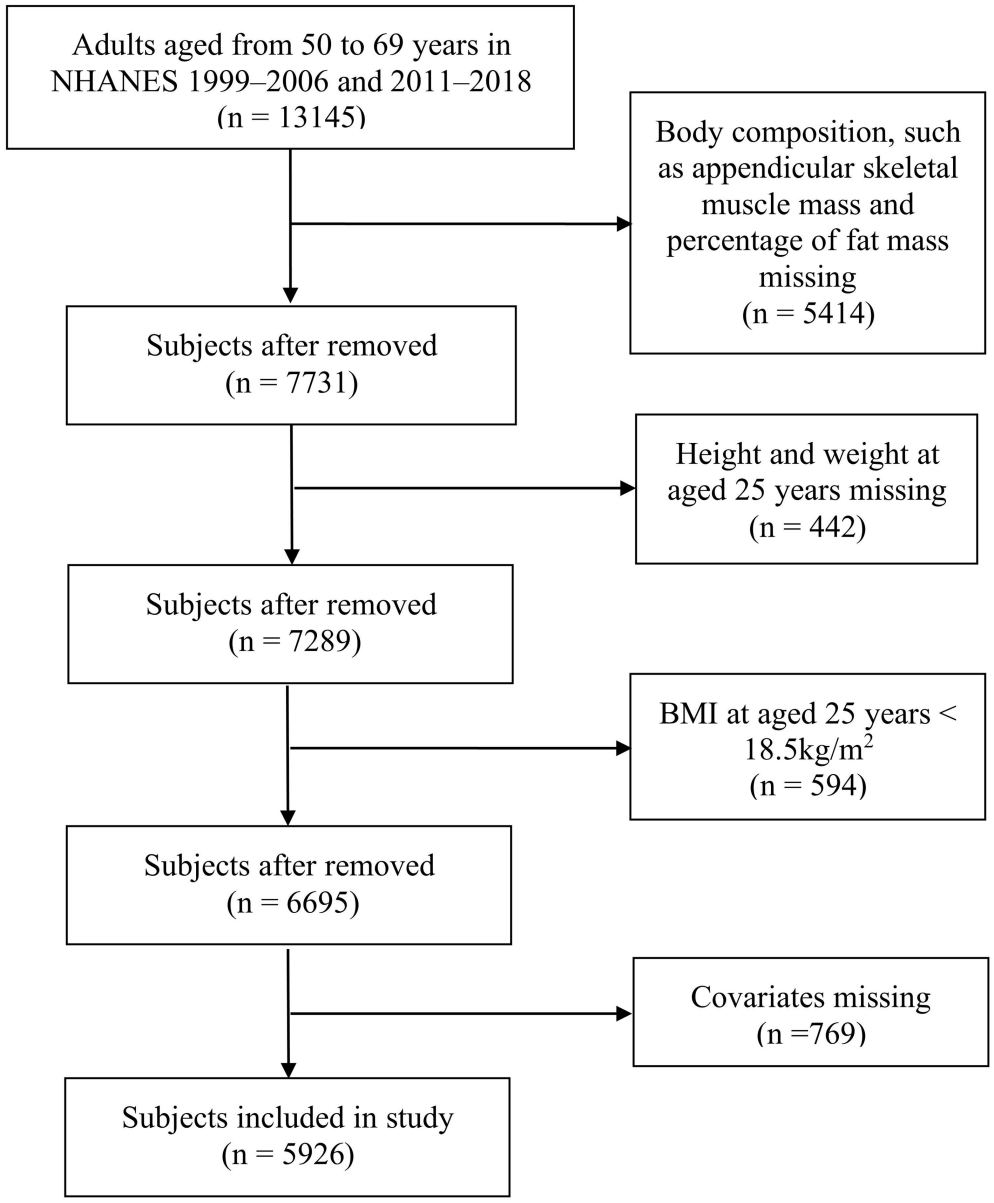
Flowchart of participant selection.

Figure 1 shows the selection process of the study. Eventually, a total of 5,926 subjects (3,031 men and 2,895 women), aged 57.7 ± 5.5 years, were enrolled in this study. Of these subjects, 411 (6.9%) had sarcopenia, 3,171 (53.5%) had obesity, and 407 (6.9%) had SO.

In men, the levels of BMI_25_, BMI, ASM, and FM (%) were higher in the overweight_25_ group and the obesity_25_ group compared to the healthy weight_25_ group (*p* < 0.05). The levels of BMI_25_, BMI, ASM, and FM (%) were higher in the obesity_25_ group compared to the overweight_25_ group (*p* < 0.05). The levels of ASM/Wt were lower in the overweight_25_ group and obesity_25_ group compared to the healthy weight_25_ group (*p* < 0.05). The levels of ASM/Wt were lower in the obesity_25_ group compared to the overweight_25_ group (*p* < 0.05) (Table 1). In women, the levels of BMI_25_, BMI, ASM, and FM (%) were higher in the overweight_25_ group and obesity_25_ group compared to the healthy weight_25_ group (*p* < 0.05). The levels of BMI_25_, BMI, and ASM were higher in the obesity_25_ group compared to the overweight_25_ group (*p* < 0.05). The levels of ASM/Wt were lower in the overweight_25_ group and obesity_25_ group compared to the healthy weight_25_ group (*p* < 0.05) (Table 2).

**Table 1:** Characteristics of men by the status of obesity at age 25 in the NHANES.

Variable	Healthy weight_25_ (*n* = 1,872)	Overweight_25_ (*n* = 946)	Obesity_25_ (*n* = 213)	*F or χ* ^2^	*p*
Age (y) mean (SD)	57.9 (5.5)	57.4 (5.6)	57.4 (5.6)	2.95	0.052
Ethnicity (%)					
Non-Hispanic White	904 (48.3)	482 (51.0)*	103 (48.4)	14.75	0.005
Non-Hispanic Black	359 (19.2)	213 (22.5)	53 (24.9)		
Other Race	609 (32.5)	251 (26.5)	57 (26.7)		
Family PIR mean (SD)	2.98 (1.67)	3.04 (1.64)	2.85 (1.62)	1.20	0.301
Smoking history					
Never	1,292 (69.0)	683 (72.2)	143 (67.2)	4.46	0.347
Former smoker	314 (16.8)	145 (15.3)	35 (16.4)		
Current smoker	266 (14.2)	118 (12.5)	35 (16.4)		
Energy (kcal) mean (SD)	2,342.9 (1,026.0)	2,348.6 (989.7)	2,358.7 (1,067.3)	0.03	0.972
Protein (g) mean (SD)	90.3 (44.3)	92.5 (42.9)	95.4 (47.5)	1.78	0.169
Physical activity (%)	1,181 (63.1)	612 (64.7)	121 (56.8)	4.65	0.098
Diabetes (%)	341 (18.2)	280 (29.6)*	100 (47.0)*#	112.70	<0.001
Hypertension (%)	804 (43.0)	488 (51.6)*	135 (63.4)*#	43.25	<0.001
Cardiovascular disease (%)	255 (13.6)	168 (17.8)*	56 (26.3)*#	27.02	<0.001
Cancer (%)	166 (8.9)	78 (8.3)	17 (8.0)	0.42	0.809
Number of prescription medicines mean (SD)	2.0 (2.5)	2.6 (3.0)*	3.3 (3.0)*#	35.92	<0.001
BMI_25_ (kg/m^2^) mean (SD)	22.2 (1.7)	26.7 (1.4)*	33.4 (3.7)*#	4,724.53	<0.001
Height (cm) mean (SD)	174.0 (7.4)	174.8 (7.2)*	175.0 (8.2)	4.83	0.008
Weight (kg) mean (SD)	82.9 (15.6)	95.1 (17.9)*	108.3 (23.4)*#	319.15	<0.001
BMI (kg/m^2^) mean (SD)	27.3 (4.6)	31.0 (5.2)*	35.3 (7.0)*#	355.68	<0.001
ASM (kg) mean (SD)	25.0 (4.0)	27.9 (4.7)*	30.3 (6.0)*#	226.13	<0.001
ASM/Wt (%) mean (SD)	30.5 (3.1)	29.6 (3.1)*	28.3 (3.2)*#	63.49	<0.001
FM (%) mean (SD)	28.2 (5.5)	30.3 (5.4)*	33.0 (6.1)*#	101.16	<0.001

NHANES, National Health and Nutrition Examination Survey; SD, standard deviation; PIR, poverty income ratio; BMI_25_, body mass index at age 25 years; BMI, body mass index at the time of survey; ASM, appendicular skeletal muscle mass; ASM/Wt, ASM divided by weight; FM (%), percentage of fat mass. The healthy weight_25_ group was defined as having a BMI_25_ < 25 kg/m^2^ and ≥18.5 kg/m^2^. The overweight_25_ group was defined as having a BMI_25_ < 30 kg/m^2^ and ≥25 kg/m^2^. The obesity_25_ group was defined as having a BMI_25_ ≥ 30 kg/m^2^. *Compared to the healthy weight_25_ group *p* < 0.05, # compared to the overweight_25_ group *p* < 0.05.

**Table 2:** Characteristics of women by the status of obesity at age 25 in the NHANES.

Variable	Healthy weight_25_ (*n* = 2,307)	Overweight_25_ (*n* = 388)	Obesity_25_ (*n* = 200)	*F or χ* ^2^	*p*
Age (y) mean (SD)	57.8 (5.5)	57.4 (5.8)	56.3 (5.1)*	7.95	<0.001
Ethnicity (%)					
Non-Hispanic White	1,155 (50.1)	142 (36.6)*	76 (38.0)*	50.01	<0.001
Non-Hispanic Black	445 (19.3)	128 (33.0)	56 (28.0)		
Other Race	707 (30.6)	118 (30.4)	68 (34.0)		
Smoking history					
Never	1,825 (79.1)	311 (80.2)	145 (72.5)	8.52	0.074
Former smoker	251 (10.9)	45 (11.6)	24 (12.0)		
Current smoker	231 (10.0)	32 (8.2)	31 (15.5)		
Family PIR mean (SD)	2.95 (1.64)	2.60 (1.63)*	2.09 (1.52)*#	30.58	<0.001
Energy (kcal) mean (SD)	1,701.0 (674.0)	1,715.5 (706.0)	1,744.6 (778.4)	0.41	0.661
Protein (g) mean (SD)	65.3 (29.3)	66.6 (32.0)	71.0 (39.9)*	3.35	0.035
Physical activity (%)	1,312 (56.9)	197 (50.8)*	87 (43.5)*	16.74	<0.001
Diabetes (%)	387 (16.8)	125 (32.2)*	95 (47.5)*#	139.06	<0.001
Hypertension (%)	1,111 (48.2)	250 (64.4)*	135 (67.5)*	56.78	<0.001
Cardiovascular disease (%)	239 (10.4)	57 (14.7)*	39 (19.5)*	19.29	<0.001
Cancer (%)	282 (12.2)	40 (10.3)	18 (9.0)	2.74	0.255
Number of prescription medicines mean (SD)	2.7 (2.9)	3.3 (3.3)*	4.0 (3.4)*#	21.51	<0.001
BMI_25_ (kg/m^2^) mean (SD)	21.4 (1.7)	26.9 (1.3)*	35.9 (6.1)*#	4,413.52	<0.001
Height (cm) mean (SD)	160.9 (6.7)	159.4 (7.5)*	159.8 (7.0)	10.41	<0.001
Weight (kg) mean (SD)	75.3 (16.9)	89.7 (20.8)*	97.6 (24.5)*#	221.31	<0.001
BMI (kg/m^2^) mean (SD)	29.0 (6.1)	35.2 (7.1)*	38.0 (8.3)*#	299.39	<0.001
ASM (kg) mean (SD)	17.4 (3.5)	20.0 (4.5)*	21.3 (5.2)*#	162.39	<0.001
ASM/Wt (%) mean (SD)	23.3 (2.6)	22.5 (2.4)*	22.0 (2.2)*	39.62	<0.001
FM (%) mean (SD)	41.4 (5.7)	44.3 (5.0)*	45.4 (5.0)*	82.96	<0.001

NHANES, National Health and Nutrition Examination Survey; SD, standard deviation; PIR, poverty income ratio; BMI_25_, body mass index at age 25; BMI, body mass index at the time of survey; ASM, appendicular skeletal muscle mass; ASM/Wt, ASM divided by weight; FM (%), percentage of fat mass. The healthy weight_25_ group was defined as having a BMI_25_ < 25 kg/m^2^ and ≥18.5 kg/m^2^. The overweight_25_ group was defined as having a BMI_25_ < 30 kg/m^2^ and ≥25 kg/m^2^. The obesity_25_ group was defined as having a BMI_25_ ≥ 30 kg/m^2^. *Compared to the healthy weight_25_ group *p* < 0.05, #compared to the overweight_25_ group *p* < 0.05.

The prevalence of SO was 5.4, 8.5, and 16.5% in the healthy weight_25_, overweight_25_, and obesity_25_ groups, respectively. The univariate logistic regression analysis (Model 1) revealed that the prevalence of SO in the overweight_25_ group and obesity_25_ group was 1.619 (95%CI: 1.280–2.047, *p* < 0.001) times and 3.448 (95%CI: 2.573–4.619, *p* < 0.001) times higher than those in the healthy weight_25_ group. After adjusting for age, gender (Men = 1, Women = 2), ethnicity (Non-Hispanic White = 0, Non-Hispanic Black = 1, and Other Race = 2), Family PIR, smoking history (Never = 0, Former smoker = 1, and Current smoker = 2), energy intake, protein intake, physical activity (No = 0 and Yes = 1), diabetes (No = 0 and Yes = 1), hypertension (No = 0 and Yes = 1), cardiovascular disease (No = 0 and Yes = 1), cancer (No = 0 and Yes = 1), and number of prescription medicines (Model 2), the prevalence of SO in the overweight_25_ and obesity_25_ groups was 1.382 (95%CI: 1.071–1.783, *p* = 0.013) times and 2.746 (95%CI: 1.987–3.795, *p* < 0.001) times higher than those in adults with the healthy weight_25_ group. After further adjusting for BMI at the time of survey (Model 3), the prevalence of SO in the overweight_25_ group and obesity_25_ group was 1.161 (95%CI: 0.898–1.500, *p* = 0.254) times and 2.286 (95%CI: 1.651–3.165, *p* < 0.001) times higher than those in the healthy weight_25_ group (all VIFs < 10, Table 3). After weighted analysis, the results were similar (Supplementary Table 1).

**Table 3:** The relationship between the status of obesity at age 25 and sarcopenic obesity among middle-aged and older adults in the United States.

Sarcopenic obesity	BMI_25_	*n* (%)	Model 1		Model 2		Model 3	
			OR (95%CI)	*p*	OR (95%CI)	*p*	OR (95%CI)	*p*
Sarcopenia	Healthy weight_25_ (*n* = 4,179)	229 (5.5)	Reference		Reference		Reference	
	Overweight_25_ (*n* = 1,334)	114 (8.6)	1.612 (1.276–2.036)	<0.001	1.378 (1.069–1.776)	0.013	1.166 (0.903–1.505)	0.238
	Obesity_25_ (*n* = 413)	68 (16.5)	3.400 (2.538–4.554)	<0.001	2.729 (1.975–3.770)	<0.001	2.288 (1.653–3.166)	<0.001
Obesity	Healthy weight_25_ (*n* = 4,179)	2,016 (48.2)	Reference		Reference		Reference	
	Overweight_25_ (*n* = 1,334)	827 (62.0)	1.750 (1.543–1.985)	<0.001	1.820 (1.589–2.085)	<0.001	1.227 (1.060–1.420)	0.006
	Obesity_25_ (*n* = 413)	328 (79.4)	4.140 (3.237–5.296)	<0.001	3.601 (2.785–4.657)	<0.001	2.505 (1.902–3.298)	<0.001
Sarcopenic obesity	Healthy weight_25_ (*n* = 4,179)	226 (5.4)	Reference		Reference		Reference	
	Overweight_25_ (*n* = 1,334)	113 (8.5)	1.619 (1.280–2.047)	<0.001	1.382 (1.071–1.783)	0.013	1.161 (0.898–1.500)	0.254
	Obesity_25_ (*n* = 413)	68 (16.5)	3.448 (2.573–4.619)	<0.001	2.746 (1.987–3.795)	<0.001	2.286 (1.651–3.165)	<0.001

Model 1: univariate logistic regression analysis. Model 2: further adjusted for age, gender (Men = 1 and Women = 2), ethnicity (Non-Hispanic White = 0, Non-Hispanic Black = 1, and Other Race = 2), Family PIR, smoking history (Never = 0, Former smoker = 1, Current smoker = 2), energy intake, protein intake, physical activity (No = 0, Yes = 1), diabetes (No = 0, Yes = 1), hypertension (No = 0, Yes = 1), cardiovascular disease (No = 0, Yes = 1), cancer (No = 0, Yes = 1), and number of prescription medicines as covariates. OR, odds ratio; CI, confidence interval; BMI, body mass index at the time of survey; BMI_25_, body mass index at age 25; PIR, poverty income ratio. The healthy weight_25_ group was defined as having a BMI_25_ < 25 kg/m^2^ and ≥18.5 kg/m^2^. The overweight_25_ group was defined as having a BMI_25_ < 30 kg/m^2^ and ≥25 kg/m^2^. The obesity_25_ group was defined as having a BMI_25_ ≥ 30 kg/m^2^.

The analysis was stratified by the level of BMI at the time of survey. In adults with BMI < 25 kg/m^2^, the prevalence of SO was 0.7, 1.9, and 0.0% in the healthy weight_25_, overweight_25_, and obesity_25_ groups, respectively. In adults with BMI ≥ 25 kg/m^2^, the prevalence of SO was 7.3, 9.1, and 17.4% in the healthy weight_25_, overweight_25_, and obesity_25_ groups, respectively. After adjusting for confounding factors, the prevalence of SO in the overweight_25_ and obesity_25_ groups was 1.149 times (95%CI: 0.886–1.489, *p* = 0.296) and 2.317 times (95%CI: 1.670–3.216, *p* < 0.001) higher than those in the healthy weight_25_ group with BMI ≥ 25 kg/m^2^ (All VIFs < 10, Table 4). After weighted analysis, the results were similar (Supplementary Table 2).

**Table 4:** The relationship between obesity status at age 25 and sarcopenic obesity among middle-aged and older adults in the United States (stratified by BMI).

Stratified by BMI	BMI_25_	*n* (%)	Model 1		Model 2	
			OR (95%CI)	*p*	OR (95%CI)	*p*
<25 kg/m^2^ (*n* = 1,339)	Healthy weight_25_ (*n* = 1,210)	9 (0.7)	Reference		Reference	
	Overweight_25_ (*n* = 107)	2 (1.9)	2.542 (0.542–11.917)	0.237	3.006 (0.533–16.965)	0.213
	Obesity_25_ (*n* = 22)	0 (0.0)	NA	NA	NA	NA
≥25 kg/m^2^ (*n* = 4,587)	Healthy weight_25_ (*n* = 2,969)	217 (7.3)	Reference		Reference	
	Overweight_25_ (*n* = 1,227)	111 (9.1)	1.261 (0.993–1.602)	0.057	1.149 (0.886–1.489)	0.296
	Obesity_25_ (*n* = 391)	68 (17.4)	2.670 (1.986–3.589)	<0.001	2.317 (1.670–3.216)	<0.001

Model 1: univariate logistic regression analysis. Model 2: further adjusted for age, gender (Men = 1, Women = 2), ethnicity (Non-Hispanic White = 0, Non-Hispanic Black = 1, and Other Race = 2), Family PIR, smoking history (Never = 0, Former smoker = 1, Current smoker = 2), energy intake, protein intake, physical activity (No = 0, Yes = 1), diabetes (No = 0, Yes = 1), hypertension (No = 0, Yes = 1), cardiovascular disease (No = 0, Yes = 1), cancer (No = 0, Yes = 1), and number of prescription medicines as covariates. OR, odds ratio; CI, confidence interval; BMI, body mass index at the time of survey; BMI_25_, body mass index at age 25; PIR, poverty income ratio. The healthy weight_25_ group was defined as having a BMI_25_ < 25 kg/m^2^ and ≥18.5 kg/m^2^. The overweight_25_ group was defined as having a BMI_25_ < 30 kg/m^2^ and ≥25 kg/m^2^. The obesity_25_ group was defined having a BMI_25_ ≥ 30 kg/m^2^.

The gender-, age-, and ethnicity-specific analyses were also conducted (Supplementary Table 3). The prevalence of SO was higher in men compared to women (8.1% vs. 5.6%, *χ*^2^ = 14.32, *p* < 0.001). Both the overweight_25_ group and the obesity_25_ group were correlated with SO in both men and women. The prevalence of SO was higher in adults aged 60–69 years compared to those aged 50–59 years (9.7% vs. 5.3%, *χ*^2^ = 41.64, *p* < 0.001). The overweight_25_ group and the obesity_25_ group were found to be correlated with SO in adults across both age ranges, 50–59 years and 60–69 years. The prevalence of SO was lower in Non-Hispanic Black adults compared to Non-Hispanic White and Other Race adults (2.2% vs. 8.0% vs. 8.3%, *χ*^2^ = 55.39, *p* < 0.001). The obesity_25_ group was found to be correlated with SO in both ethnicities. However, the overweight_25_ group was found to be correlated with SO only in Non-Hispanic White adults and Other Race adults.

The time required to complete a 20-foot walk was higher in the obesity_25_ group compared to the healthy weight_25_ group in the 1999–2002 cycles (*p* < 0.05). In the 2011–2014 cycles, the levels of combined grip strength were higher in the overweight_25_ group compared to the healthy weight_25_ group and obesity_25_ group (*p* < 0.05). The levels of MQI were lower in the overweight_25_ group and obesity_25_ group compared to the healthy weight_25_ group (*p* < 0.05). The levels of MQI were lower in the obesity_25_ group compared to the overweight_25_ group (*p* < 0.05) (Table 5).

**Table 5:** Muscle function based on obesity status at age 25 in the NHANES.

Cycles	Variable	Healthy weight_25_	Overweight_25_	Obesity_25_	*F*	*p*
1999–2002	*N*	1,358	375	101		
	ASM (kg) mean (SD)	20.9 (5.5)	25.6 (5.5)*	26.2 (6.9)*	133.33	<0.001
	Time to complete 20-foot walk (s) mean (SD)	6.17 (1.94)	6.31 (1.79)	6.68 (1.81)*	3.84	0.022
2011–2014	*N*	662	234	88		
	ASM (kg) mean (SD)	21.0 (5.2)	26.5 (6.3)*	27.2 (7.4)*	107.99	<0.001
	Combined grip strength (kg) mean (SD)	69.4 (19.5)	80.7 (20.2)*	71.8 (20.8)#	28.32	<0.001
	MQI (kg/kg) mean (SD)	3.33 (0.59)	3.08 (0.54)*	2.69 (0.63)*#	55.69	<0.001

NHANES, National Health and Nutrition Examination Survey; SD, standard deviation; BMI_25_, body mass index at age 25; ASM, appendicular skeletal muscle mass; MQI, muscle quality index. The healthy weight_25_ group was defined as having a BMI_25_ < 25 kg/m^2^ and ≥18.5 kg/m^2^. The overweight_25_ group was defined as having BMI_25_ < 30 kg/m^2^ and ≥25 kg/m^2^. The obesity_25_ group was defined as having a BMI_25_ ≥ 30 kg/m^2^. *Compared to the healthy weight_25_ group *p* < 0.05, #compared to the overweight_25_ group *p* < 0.05.

## Discussion

The present study found that the prevalence of SO among middle-aged and older adults in the United States was 6.9%. This estimate is comparable to a previously published meta-analysis, which reported a global prevalence of 11% in older adults (23). Notably, there is considerable variability in the reported prevalence of SO, largely due to the absence of universally accepted diagnostic criteria. In this study, the diagnostic criteria were based on the joint guidelines of the European Society for Clinical Nutrition and Metabolism (ESPEN) and the European Association for the Study of Obesity (EASO) (18), and it also incorporated age-, gender-, and ethnicity-specific cutoffs (19, 21).

Our findings indicate that the prevalence of SO increased with higher obesity status in early adulthood. Previous epidemiological studies have demonstrated a significant trajectory effect of obesity over the life course (10, 24). Consistent with these findings, our results indicate that individuals who were overweight or obese at age 25 had higher BMI levels during middle and old age. After adjusting for overweight and obesity status at the time of survey, early adulthood obesity remained significantly associated with SO in later life, indicating an independent effect of early obesity on the development of SO.

Obesity in early adulthood has also been strongly associated with reduced muscle mass in young adults (25). In our analysis of SO components, early adulthood obesity remained independently associated with both sarcopenia and obesity in middle-aged and older adults, even after controlling for concurrent obesity status. Although individuals who were obese in early adulthood may have higher absolute muscle mass in middle and old age compared to those with normal weight, their relative muscle mass remains lower.

Furthermore, early adulthood obesity was associated with reduced muscle function in middle-aged and older individuals. Despite higher absolute muscle mass, individuals with early adulthood obesity exhibited slower walking speeds and comparable grip strength to those with a healthy weight in early adulthood. Muscle quality index (MQI), defined as the ratio of muscle strength to muscle mass, is used to evaluate muscle quality (22). Our findings indicate that MQI decreased with increasing early adulthood obesity status. These results align with prior findings indicating that prolonged exposure to obesity is linked to poorer muscle strength in later life (26).

Aging is associated with significant alterations in body composition. Body fat tends to increase until approximately the seventh decade of life and subsequently declines. In contrast, muscle mass begins to decrease after peaking in the fourth decade, leading to weight gain predominantly in the form of fat rather than lean mass (27). Obesity may exacerbate these changes through several mechanisms.

Inflammatory pathways: Obesity activates macrophages, thereby inducing low-grade chronic inflammation that contributes to insulin resistance. This cascade promotes a decline in both muscle mass and function (26, 27).

Ectopic fat deposition: Obesity facilitates the deposition of fat in ectopic locations, including skeletal muscle. An increased level of intramyocellular lipids has been associated with impaired muscle function in obese older adults (28).

Adipocytokine dysregulation: Adipose tissue functions as an endocrine organ, secreting various adipokines such as leptin and adiponectin. Obesity disrupts the normal secretion patterns of these adipokines. Elevated levels of leptin, indicative of leptin resistance, are closely associated with decreased muscle mass in middle-aged and older adults (29, 30).

Changes in obesity status may influence the risk of developing sarcopenic obesity in middle-aged and older adults. Among individuals who were overweight or obese in early adulthood, those whose BMI decreased to below 25 kg/m^2^ in later life had a significantly lower prevalence of SO compared to those whose BMI remained above 25 kg/m^2^. Moreover, individuals with persistently high BMI values throughout life had a higher prevalence of SO than those who became overweight or obese at a later stage.

Two large-scale prospective cohort studies have also reported a strong association between age and obesity-related outcomes. Compared to individuals with normal weight, obesity in younger adults is linked to a higher risk of all-cause mortality, whereas obesity in older adults does not appear to increase mortality risk (31). These findings suggest that implementing weight management strategies among overweight and obese young adults may have potential clinical benefits in reducing the risk of SO in later life. However, these implications should be validated through prospective cohort studies or randomized clinical trials.

This study has several strengths. It included a large sample size and conducted stratified analyses based on gender, age, and ethnicity. Across all gender and age categories, overweight and obesity in early adulthood were associated with an elevated risk of SO in middle-aged and older individuals. However, ethnic differences were observed in the association between early adulthood overweight status and SO among Non-Hispanic Black individuals and other racial groups. The prevalence of SO was lower among Non-Hispanic Black participants. Specifically, in this population, the prevalence of SO among those who were overweight in early adulthood was comparable to that observed in those with a healthy weight at the same age. Although residual confounding may be present, these findings may be partially explained by ethnic differences in body composition observed in the NHANES population. When BMI is held constant, Non-Hispanic Black individuals tend to have greater muscle mass and lower fat mass compared to other ethnicities (32).

Nevertheless, several limitations should be acknowledged. First, sarcopenia in this study was assessed solely based on skeletal muscle mass, as measurements of muscle strength and function—such as grip strength and walking speed—were only available in a subset of the NHANES cycles. Recent consensus definitions emphasize muscle strength and physical function as core components of sarcopenia (33). Although walking speed, grip strength, and the muscle quality index were analyzed in a subsample, the exclusion of functional metrics from the core SO definition may have led to misclassification. Hence, future research is warranted to confirm the association between early adulthood obesity and sarcopenic obesity—defined simultaneously by muscle mass, strength, and function—in middle-aged and older adults.

Second, early adulthood overweight and obesity were determined using self-reported height and weight at age 25. Although self-reported anthropometric data are widely used in epidemiological studies (16, 34), the accuracy of recalled weight over extended periods may be affected by recall bias (35, 36). While this approach is more economical and convenient than prospective tracking, the long recall interval could compromise the precision of the data. Recall accuracy is known to differ by age, with older adults (≥60 years) generally having lower accuracy compared to younger individuals (<60 years) (37). However, subgroup analyses stratified by age group (50–59 years and 60–69 years) showed similar results, supporting the robustness of the findings.

Third, the retrospective design of this study limits causal inference. The relationship between early adulthood obesity and subsequent SO should be further investigated through well-designed prospective cohort studies. Moreover, potential residual confounding factors—particularly those related to physical activity, comorbidities, or early-life dietary patterns—were not controlled for in this study.

In conclusion, this study demonstrates that obesity in early adulthood is strongly associated with an increased risk of sarcopenic obesity in middle-aged and older individuals. Weight changes in early adulthood exert a potential influence on the likelihood of developing SO later in life. Restoration of body weight to a normal range may reduce the risk of SO, whereas persistent overweight or obesity may elevate this risk. These findings underscore the importance of early life weight management as a potential strategy for preventing SO in old age.

## Data Availability

The raw data supporting the conclusions of this article will be made available by the authors without undue reservation.

## Footnotes

^1^https://www.cdc.gov/nchs/nhanes/about/erb.html?CDC_AAref_Val=; https://www.cdc.gov/nchs/nhanes/irba98.htm

^2^ https://www.cdc.gov/bmi/adult-calculator/bmi-categories.html
