# Supplementary material for: The relationship between early adulthood obesity and sarcopenic obesity among middle-aged and older adults in the United States
Source: Front Public Health. 2025 Sep 1;13:1609325. doi: 10.3389/fpubh.2025.1609325 (PMC12433845; doi:10.3389/fpubh.2025.1609325)
Supplement: Supplementary file 1 [file Table_1.DOC]

Supplementary Table 1 The relationship between the status of obesity at aged 25 years and sarcopenic obesity among middle-aged and elderly adults in the United States (Weighted).

| Sarcopenic obesity | BMI_25_ | n | Unweighted (%) | Weighted (%) | Model 1 | | Model 2 | | Model 3 | |
| --- | --- | --- | --- | --- | --- | --- | --- | --- | --- | --- |
|  |  |  |  |  | OR(95%CI) | *P* | OR(95%CI) | *P* | OR(95%CI) | *P* |
| Sarcopenia | Healthy weight_25_  (n=4179) | 229 | 5.5 | 4.6 | Reference |  | Reference |  | Reference |  |
|  | Overweight_25_  (n=1334) | 114 | 8.6 | 8.4 | 1.921(1.403～2.631) | <0.001 | 1.541(1.088～2.183) | 0.015 | 1.275(0.906～1.794) | 0.162 |
|  | Obesity_25_  (n=413) | 68 | 16.5 | 18.6 | 4.801(3.461～6.661) | <0.001 | 3.431(2.345～5.019) | <0.001 | 2.781(1.908～4.052) | <0.001 |
| Obesity | Healthy weight_25_  (n=4179) | 2016 | 48.2 | 47.8 | Reference |  | Reference |  | Reference |  |
|  | Overweight_25_  (n=1334) | 827 | 62.0 | 63.7 | 1.914(1.606～2.281) | <0.001 | 1.992(1.657～2.394) | <0.001 | 1.291(1.063～1.568) | 0.011 |
|  | Obesity_25_  (n=413) | 328 | 79.4 | 82.7 | 5.212(3.795～7.157) | <0.001 | 4.392(3.194～6.041) | <0.001 | 2.842(2.032～3.975) | <0.001 |
| Sarcopenic obesity | Healthy weight_25_  (n=4179) | 226 | 5.4 | 4.5 | Reference |  | Reference |  | Reference |  |
|  | Overweight_25_  (n=1334) | 113 | 8.5 | 8.3 | 1.909(1.386～2.629) | <0.001 | 1.525(1.072～2.167) | 0.019 | 1.255(0.889～1.771) | 0.196 |
|  | Obesity_25_  (n=413) | 68 | 16.5 | 18.6 | 4.827(3.478～6.700) | <0.001 | 3.423(2.331～5.026) | <0.001 | 2.761(1.883～4.049) | <0.001 |

Model 1: univariate logistic regression analysis. Model 2: further adjusted for age, gender (Males = 1, Females = 2), race (Non-Hispanic White = 0, Non-Hispanic Black = 1, Other Race = 2), Family PIR, smoking history (Never = 0, Former smoker = 1, Current smoker = 2), energy intake, protein intake, physical activity (No = 0, Yes = 1), diabetes (No = 0, Yes = 1), hypertension (No = 0, Yes = 1), cardiovascular disease (No = 0, Yes = 1), cancer (No = 0, Yes = 1) and, number of prescription medicines as covariates. OR: odds ratio; CI: confidence interval; BMI: body mass index at the time of survey; BMI_25_: body mass index at aged 25 years; PIR: poverty income ratio. Healthy weight_25_ group was defined as BMI_25_ < 25 kg/m^2^ and ≥18.5 kg/m^2^. Overweight_25_ group was defined as BMI_25_ < 30 kg/m^2^ and ≥25 kg/m^2^. Obesity_25_ group was defined as BMI_25_ ≥30 kg/m^2^.

Supplementary Table 2 The relationship between the status of obesity at aged 25 years and sarcopenic obesity among middle-aged and elderly adults in the United States (stratified by BMI, Weighted).

| Stratified by BMI | BMI_25_ | n | Unweighted (%) | Weighted (%) | Model 1 | | Model 2 | |
| --- | --- | --- | --- | --- | --- | --- | --- | --- |
|  |  |  |  |  | OR(95%CI) | *P* | OR(95%CI) | *P* |
| < 25 kg/m^2^  (n=1339) | Healthy weight_25_  (n=1210) | 9 | 0.7 | 0.7 | Reference |  | Reference |  |
|  | Overweight_25_  (n=107) | 2 | 1.9 | 2.9 | 4.183(0.758～23.070) | 0.100 | 2.750(0.223～33.888) | 0.426 |
|  | Obesity_25_  (n=22) | 0 | 0.0 | 0.0 | NA | NA | NA | NA |
| ≥25 kg/m^2^  (n=4587) | Healthy weight_25_  (n=2969) | 217 | 7.3 | 6.2 | Reference |  | Reference |  |
|  | Overweight_25_  (n=1227) | 111 | 9.1 | 8.7 | 1.436(1.045～1.972) | 0.026 | 1.230(0.867～1.745) | 0.243 |
|  | Obesity_25_  (n=391) | 68 | 17.4 | 19.3 | 3.615(2.602～5.021) | <0.001 | 2.816(1.930～4.109) | <0.001 |

Model 1: univariate logistic regression analysis. Model 2: further adjusted for age, gender (Males = 1, Females = 2), race (Non-Hispanic White = 0, Non-Hispanic Black = 1, Other Race = 2), Family PIR, smoking history (Never = 0, Former smoker = 1, Current smoker = 2), energy intake, protein intake, physical activity (No = 0, Yes = 1), diabetes (No = 0, Yes = 1), hypertension (No = 0, Yes = 1), cardiovascular disease (No = 0, Yes = 1), cancer (No = 0, Yes = 1) and, number of prescription medicines as covariates. OR: odds ratio; CI: confidence interval; BMI: body mass index at the time of survey; BMI_25_: body mass index at aged 25 years; PIR: poverty income ratio. Healthy weight_25_ group was defined as BMI_25_ < 25 kg/m^2^ and ≥18.5 kg/m^2^. Overweight_25_ group was defined as BMI_25_ < 30 kg/m^2^ and ≥25 kg/m^2^. Obesity_25_ group was defined as BMI_25_ ≥30 kg/m^2^.

Supplementary Table 3 The relationship between the status of obesity at aged 25 years and sarcopenic obesity among middle-aged and elderly adults in the United States (stratified by gender, age, and race).

| Stratified by gender | BMI_25_ | n(%) | OR(95%CI) | *P* |
| --- | --- | --- | --- | --- |
| Males  (n=3031) | Healthy weight_25_  (n=1872) | 115(6.1) | Reference |  |
|  | Overweight_25_  (n=946) | 85(9.0) | 1.508(1.126～2.020) | 0.006 |
|  | Obesity_25_  (n=213) | 45(21.1) | 4.092(2.801～5.980) | <0.001 |
| Females  (n=2895) | Healthy weight_25_  (n=2307) | 111(4.8) | Reference |  |
|  | Overweight_25_  (n=388) | 28(7.2) | 1.539(1.002～2.364) | 0.049 |
|  | Obesity_25_  (n=200) | 23(11.5) | 2.571(1.600～4.131) | <0.001 |
| Stratified by age | BMI_25_ | n(%) | OR(95%CI) | *P* |
| 50-59 years  (n=3817) | Healthy weight_25_  (n=2642) | 99(3.8) | Reference |  |
|  | Overweight_25_  (n=887) | 61(6.9) | 1.897(1.366～2.635) | <0.001 |
|  | Obesity_25_  (n=288) | 42(14.6) | 4.386(2.987～6.438) | <0.001 |
| 60-69 years  (n=2109) | Healthy weight_25_  (n=1537) | 127(8.3) | Reference |  |
|  | Overweight_25_  (n=447) | 52(11.6) | 1.462(1.039～2.056) | 0.029 |
|  | Obesity_25_  (n=125) | 26(20.8) | 2.916(1.825～4.658) | <0.001 |
| Stratified by race | BMI_25_ | n(%) | OR(95%CI) | *P* |
| Non-Hispanic White  (n=2862) | Healthy weight_25_  (n=2059) | 126(6.1) | Reference |  |
|  | Overweight_25_  (n=624) | 67(10.7) | 1.845(1.352～2.518) | <0.001 |
|  | Obesity_25_  (n=179) | 37(20.7) | 3.997(2.668～5.988) | <0.001 |
| Non-Hispanic Black  (n=1254) | Healthy weight_25_  (n=804) | 11(1.4) | Reference |  |
|  | Overweight_25_  (n=341) | 8(2.4) | 1.732(0.690～4.344) | 0.242 |
|  | Obesity_25_  (n=109) | 8(7.3) | 5.710(2.244～14.530) | <0.001 |
| Other Race  (n=1810) | Healthy weight_25_  (n=1316) | 89(6.8) | Reference |  |
|  | Overweight_25_  (n=369) | 38(10.3) | 1.583(1.062～2.358) | 0.024 |
|  | Obesity_25_  (n=125) | 23(18.4) | 3.109(1.884～5.130) | <0.001 |

Univariate logistic regression analysis. OR: odds ratio; CI: confidence interval; BMI_25_: body mass index at aged 25 years. Healthy weight_25_ group was defined as BMI_25_ < 25 kg/m^2^ and ≥18.5 kg/m^2^. Overweight_25_ group was defined as BMI_25_ < 30 kg/m^2^ and ≥25 kg/m^2^. Obesity_25_ group was defined as BMI_25_ ≥30 kg/m^2^.
